# Editorial: Emotional intelligence in educational psychology: enhancing learning and development

**DOI:** 10.3389/fpsyg.2026.1847756

**Published:** 2026-05-29

**Authors:** Isabel Mercader Rubio, Nieves Gutiérrez Ángel, Sónia Brito-Costa

**Affiliations:** 1 Department of Psychology, University of Almeria, Almería, Spain; 2 Instituto Politecnico de Coimbra, Coimbra, Portugal

**Keywords:** educational setting, teacher training, emotional skills, innovation, emotional intelligence, motivation, pedagogical practices, mental health

Recent educational research has shown a growing interest in the role of emotional and psychological factors within the teaching and learning process. Specifically, emotional intelligence has become a key element in understanding how teachers and students face academic challenges, manage their emotions, and develop skills that promote wellbeing and performance. The analyzed articles approach this phenomenon from different perspectives, including teaching behavior, faculty wellbeing, student learning, the impact of educational technology, and socio-emotional development in diverse educational contexts.

## Emotional intelligence and teaching practice

One of the central themes is the relationship between emotional intelligence and teaching practice. A study on innovative teaching behavior in pre-service music teachers analyzes how psychological empowerment and professional commitment influence the ability of future educators to adopt innovative pedagogical practices. The results indicate that emotional intelligence facilitates emotional regulation, improves self-confidence, and fosters creativity in teaching. In this sense, psychological empowerment allows teachers to feel capable of making autonomous pedagogical decisions, while professional commitment reinforces their motivation to implement novel methodologies in the classroom (Jiang and Tong).

## Faculty wellbeing

Emotional intelligence has also been analyzed as a fundamental factor for the wellbeing of university faculty. A study focused on teachers at Chinese universities explores how this competence influences their psychological wellbeing and their ability to balance work and family responsibilities. The results show that professors with higher levels of emotional intelligence perceive greater support for work-life balance, which reduces stress and increases job satisfaction. These findings suggest that educational institutions should promote reconciliation policies and socio-emotional development programs that contribute to improving the quality of life of the faculty (Li et al.).

## Impact on students and academic performance

Regarding the student body, several studies analyze the impact of emotional intelligence on academic performance and personal development. A study based on structural equation modeling demonstrates that emotional intelligence acts as a predictor of performance in higher education. According to the results, students with a greater capacity to understand and regulate their emotions present higher levels of motivation, self-regulation, and engagement with learning. These skills facilitate persistence in the face of academic difficulties and promote more effective study strategies (Suson et al.).

Another work examines the relationship between emotional intelligence and the holistic development of students in vocational university English programs. The results indicate that emotional skills positively influence self-efficacy, social skills, and adaptation to the educational environment. In language learning contexts, where communication and social interaction are essential, emotional intelligence helps students overcome linguistic anxiety and participate actively in learning activities (Xuan).

## Educational technology and mindfulness

The incorporation of educational technologies also appears as a relevant topic. In particular, research on growth mindset in AI-mediated second language learning analyzes how the perceived usability of technological tools and the presence of generative chatbots influence student motivation. The results show that when students perceive these tools as useful and easy to use, they develop a growth mindset that favors perseverance and continuous improvement. Interaction with chatbots allows for practicing communication skills in a less intimidating environment, which reduces anxiety and encourages participation (Huang et al.).

Furthermore, another study examines the role of mindfulness as a mechanism connecting emotional intelligence with academic engagement. The results indicate that students who practice mindfulness present a greater capacity for concentration and more frequently experience flow states during learning. These states are characterized by deep involvement in the task and a sense of intrinsic satisfaction, which contributes to increasing academic commitment and motivation to learn (Wu and Ma).

## Conceptual reviews and innovative resources

In addition to empirical studies, some articles offer conceptual reviews. A theoretical review analyzes different models explaining how emotional skills influence the relationship between teachers and students, classroom management, and the educational climate. According to this review, educators who develop emotional competencies are capable of creating more positive learning environments, promoting empathy, and managing conflicts constructively. Consequently, the development of emotional intelligence should be considered an essential part of teacher training (Matjie).

Emotional education can also be addressed through innovative pedagogical resources. One study proposes the use of cinema as a tool to foster emotional intelligence in the classroom. Through the analysis of film scenes, students can explore different emotions, understand diverse perspectives, and reflect on complex social situations. This methodology favors the development of empathy and the ability to interpret emotions in real contexts (Thomas and Manalil).

## Inclusion, wellbeing, and leadership

Another group of research focuses on wellbeing and educational inclusion. A study on university students with disabilities analyzes how emotional intelligence influences their quality of life and academic success. The results show that emotional skills help students manage stress, develop support networks, and adapt to the university environment. These competencies contribute to reducing psychological barriers and facilitate full participation in academic life (Thomas and Manalil).

Teacher leadership has also been identified as a relevant factor for student wellbeing. A study conducted in Chinese universities analyzes how the leadership style of the faculty influences students' academic burnout. The results indicate that teachers who support autonomy, foster competence, and promote positive relationships significantly reduce the risk of academic burnout (Fang).

In the field of teacher wellbeing, a systematic review analyzes different psychological interventions aimed at improving the emotional competence and mental health of the faculty. Among the most effective strategies are mindfulness programs, emotional regulation training, and professional support networks. These interventions contribute to reducing occupational stress and improving satisfaction with the teaching profession (Cervellione et al.).

## Cognitive variables and feedback

The influence of positive emotions has also been a subject of study. Research on the effects of happiness and hope demonstrates that these emotions can improve executive functions such as working memory, planning, and inhibitory control. These cognitive capacities are fundamental for learning and problem-solving (Lautenbach).

Other studies explore psychological variables related to academic performance. A work on predictors of performance highlights the role of personality, rational beliefs, and self-efficacy. Results show that students with greater confidence in their abilities and traits of responsibility tend to obtain better academic results (Coşa and Cernat).

Likewise, the way academic feedback is provided can have important emotional consequences. A study demonstrates that poorly communicated feedback can generate anxiety, demotivation, and a decrease in academic engagement. Conversely, constructive feedback can strengthen motivation and learning (Tang et al.).

## Resilience and social dynamics

Socio-emotional development is also related to variables such as resilience and prosociality. One study indicates that these characteristics strengthen teaching self-efficacy and help teachers cope with classroom challenges. Similarly, research on classroom silence shows that social anxiety can limit student participation, underlining the importance of promoting positive interpersonal relationships (Mieres-Chacaltana et al.).

In broader educational contexts, some studies analyze the impact of school support on students' cognitive engagement, especially in crisis situations. Results suggest that institutional support strengthens psychological resilience and academic motivation (Wang et al.). On the other hand, methodological research has developed instruments to measure emotional competencies, such as the validation of a scale to assess the management of emotions in others among Spanish adolescents (Martín-Laguna et al.).

## Hybrid and online environments

Maintaining student engagement in hybrid/online courses is fundamental for learning and retention. The psychological empowerment of the student fosters involvement and a sense of belonging, which in turn drives class loyalty and course evaluation. The results of this research support the chain of empowerment ownership/participation → loyalty → evaluation, highlighting the need for pedagogies that foster autonomy and ownership to maintain commitment and positive course ratings in digital learning environments (Fei et al.).

## General conclusion

Taken together, the analyzed studies show that emotional intelligence plays a fundamental role in various areas of the educational field, such as faculty wellbeing, academic performance, student motivation, teaching innovation, and mental health. Furthermore, research indicates that factors such as resilience, self-efficacy, motivation, and institutional backing are related to emotional skills and contribute to academic outcomes. These findings underscore the importance of incorporating socio-emotional development into educational policies, teacher training, and pedagogical practices to foster healthier, more inclusive, and effective learning environments.

